# SeqNLS: Nuclear Localization Signal Prediction Based on Frequent Pattern Mining and Linear Motif Scoring

**DOI:** 10.1371/journal.pone.0076864

**Published:** 2013-10-29

**Authors:** Jhih-rong Lin, Jianjun Hu

**Affiliations:** Department of Computer Science and Engineering, University of South Carolina, Columbia, South Carolina, United States of America; University of Westminster, United Kingdom

## Abstract

Nuclear localization signals (NLSs) are stretches of residues in proteins mediating their importing into the nucleus. NLSs are known to have diverse patterns, of which only a limited number are covered by currently known NLS motifs. Here we propose a sequential pattern mining algorithm SeqNLS to effectively identify potential NLS patterns without being constrained by the limitation of current knowledge of NLSs. The extracted frequent sequential patterns are used to predict NLS candidates which are then filtered by a linear motif-scoring scheme based on predicted sequence disorder and by the relatively local conservation (IRLC) based masking.

The experiment results on the newly curated Yeast and Hybrid datasets show that SeqNLS is effective in detecting potential NLSs. The performance comparison between SeqNLS with and without the linear motif scoring shows that linear motif features are highly complementary to sequence features in discerning NLSs. For the two independent datasets, our SeqNLS not only can consistently find over 50% of NLSs with prediction precision of at least 0.7, but also outperforms other state-of-the-art NLS prediction methods in terms of F1 score or prediction precision with similar or higher recall rates. The web server of the SeqNLS algorithm is available at http://mleg.cse.sc.edu/seqNLS.

## Introduction

A nuclear localization signal is a protein peptide bound to carrier proteins for trafficking nuclear proteins into the nucleus. As the most direct evidence for nuclear localization, identification of NLSs can help to elucidate protein functions. However, experimental identification of such signals is costly and currently only a limited number of NLSs have been identified. It is thus desirable to develop algorithms for computational prediction of NLSs. Several NLS prediction methods have been developed such as PSORT II [1], PredictNLS [2], NLStradamus [3], cNLS Mapper [4], and NucImport [5]. PSORT II predicts NLSs based on sequence patterns implemented as three simple rules according to the classification of NLSs [6]; the rules are mainly clusters of basic amino acids K and R and gaps between the clusters. PredictNLS predicts NLSs based on 194 potential NLS motifs, which are derived from 114 experimentally verified NLSs with a silico mutagenesis approach. Nguyen Ba et. al. [3] found that NLSs tend to have similar residue frequency distributions which are different from that of background residues. Their NLStradamus algorithm detects NLSs by using a simple two-state or four-state HMMs to accommodate the frequency variations. cNLS Mapper estimates classical NLS (cNLS) functionality of a peptide by calculating sum of the functional contribution of each residue in the peptide according to the activity-based profiles, which are obtained from the systematic amino acid-replacement analyses in budding yeast. NucImport [5] builds a Bayesian network to predict nuclear localization by incorporating various attributes related to the nuclear importing. If a protein is predicted as a nuclear protein, the location of its NLS is predicted as the segment in the protein with the highest cNLS score in the inferred cNLS class based on the dependencies with other attributes in the Bayesian network.

These five NLS prediction methods have achieved different degrees of success. However, their prediction performance is still far from being sufficient to assist biologists to discover putative NLSs in protein sequences of interest. Each of them has their weakness. Although a great portion of NLSs can be covered by the rules used in PSORT II to detect NLS, quite a few patterns covered by the rules exist in peptides which do not contain NLSs, leading to a high false positive rate or low prediction precision. The sensitivity of the PredictNLS algorithm depends on the number of NLS motifs it used, which has been extended by introducing the potential NLS motifs generated using in-silico mutagenesis analysis. But they are still too specific and couldn't effectively accommodate NLS variability [3]. The performance of the NLStradamus algorithm depends on its assumption that NLSs have certain residue distributions. However, many NLS instances in our testing datasets have shown very different residue frequencies. Both cNLS mapper and NucImport algorithms are developed based on the characteristics of cNLS. However, approximately 43% of proteins localized to the nucleus may use other transport mechanisms other than the classical nuclear import pathway according to Lange et al [7].

One of the challenges of NLS prediction is that functional NLSs are not defined [8]. Many NLSs are short peptides that occur regularly in non-nuclear proteins. In fact, NLS is one type of linear motifs as defined in the database of eukaryotic linear motifs [9]. Linear motifs are short stretches of residues which are highly involved in cell signaling and regulating. To adapt to the fast fine-tuning cell regulatory process, certain characteristics of linear motifs have thus evolved and might have contributed to NLS variability: only a few residues within a linear motif are functionally important, and mutation of a single residue can switch on/off the functionality [10], [11]. The nature of shortness, flexibility and sensibility provides linear motifs evolutionary plasticity to form a functional unit and fine-tune cell singling network over short evolutionary distances, which, however, increases the difficulties in computational identification of linear motifs such as NLSs.

In the past decade, many computational approaches have been proposed to discover linear motifs. There are two categories of the methods [10]: one is supervised methods aiming to identify new instances of known linear motifs in protein sequences [9], [12], [13], [14], [15], [16], [17], [18]; the other is de novo methods for discovering new linear motifs [19], [20], [21], [22]. The challenge of the former is to discriminate between true and false positive matches. Most of such prediction algorithms take advantage of the special attributes of linear motifs [23] to remove false positive matches that are unlikely to be functional linear motifs. The latter de novo linear motif discovery algorithms [19], [20], [22] are usually based on the enrichment analysis of candidate motifs integrated with disorder prediction and evolutionary conservation. Since NLS is one type of linear motifs, the framework of the first category may apply to predicting NLSs. However, despite the availability of a number of NLS motifs [24], [25], they are either too specific [3] or they only target a specific pathway of NLSs. To cover more NLSs, we need a new approach to utilize linear motif attributes.

In this paper, we propose a novel algorithm for NLS prediction based on sequential-pattern mining and linear motif scoring. Our strategy is first to detect potential NLS candidates using the sequential-pattern mining method, which are then scored in terms of their likelihood of being (part of) NLS based on their sequence and linear motif features. The qualified candidate motifs will then be combined into NLS predictions.

## Materials and Methods

### Training and Testing dataset

We used 114 experimentally determined NLSs from NLSdb [7] as the source of the positive training dataset for sequential pattern mining. Two NLSs without a specific form in amino acid sequence and a reference citation were removed. 94 out of 112 were real NLSs of which the parent proteins could be found, while the rest 18 were either synthetic NLSs or NLSs of which the parent proteins couldn't be found. We then removed the redundant NLSs in order to avoid non-functional residues being enriched in the positive training dataset: given a NLS A, the redundant NLSs to A are defined as NLSs whose parent proteins are highly homologous to the parent protein of NLS A and are overlapped with NLS A in the alignment of their parent proteins. To remove redundant NLSs, Blastclust with 90% sequence identity and 90% sequence coverage was applied on the parent proteins of the 94 NLSs. If multiple NLSs were overlapped in the alignment of their parent proteins which were in the same cluster, then only one of the NLSs was kept; 4 out of the 94 NLSs were thus removed. In the end, 108 experimentally verified NLSs were left in our positive training dataset for sequential-pattern mining. We then collected 2238 non-nuclear proteins from the BaCello dataset [26], from which 26772 non-overlapped peptides of length 40 were randomly sampled for the negative training dataset for sequential-pattern mining. The length 40 was determined because it approximated the longest NLSs in the positive training dataset. To prepare the training dataset for linear motif scoring (to be defined below), the 90 NLSs with known parent proteins used in the training dataset of sequential-pattern mining were used as the positive training dataset. For each of the 90 NLSs, a random amino acid segment of the same length in the same parent protein which was not overlapped with any annotated NLS was collected to produce the negative dataset.

We prepared two independent testing datasets according to the species of the NLS source proteins for evaluating the NLS predictors: 1) The Yeast NLS dataset; 2) The Hybrid NLS dataset of which the parent proteins are from different species. The Yeast dataset was prepared based on the dataset used in NLStradamus [3]. The Hybrid dataset was collected by searching annotated NLSs from literature published after 2010. All NLSs in the testing datasets redundant to NLSs in the training dataset (90 NLSs with known parent proteins) were removed, and redundant NLSs in the testing dataset itself were also removed. In the end, the Yeast dataset contains 50 NLSs from 41 proteins, and the Hybrid dataset contains 73 NLSs from 53 proteins. Both datasets are provided in the supplementary file (Table S1 and Table S2).

### Overview of the proposed algorithm

Our SeqNLS algorithm is developed based on the following observations of NLSs: 1) most known NLSs are composed of a sequence of well-conserved segments of amino acids with variable-length gaps. This is because a set of NLSs binding to the same binding pockets usually share such patterns due to the geometrical or physical-interaction constraints at the binding interface. Such sequential patterns are thus over-represented among these NLSs; 2) similar to other linear motifs, NLSs usually occur in the disordered regions of the protein sequences; 3) NLSs for different pathways may be different. Our algorithm for NLS prediction can be divided into two steps: 1) mining NLS sequence patterns from experimentally verified NLS instances and then predicting NLS candidates on query sequence(s); 2) scoring candidate NLSs based on sequence and linear motif scoring and applying local conservation masking. Our sequential-pattern mining method is motivated by the fact that diversity among the experimentally verified NLSs has hampered the discovery of NLS motifs due to a limited number of NLS instances [25], [26]. SeqNLS addresses this issue by using a more general motif model: the sequential patterns.

### Sequential-pattern based prediction of NLSs

In our method, sequential pattern mining is used to extensively collect potential NLS segments/building blocks, which are then used to detect potential NLS segments in query sequences.

#### NLS sequential-pattern mining

**Figure 1:**
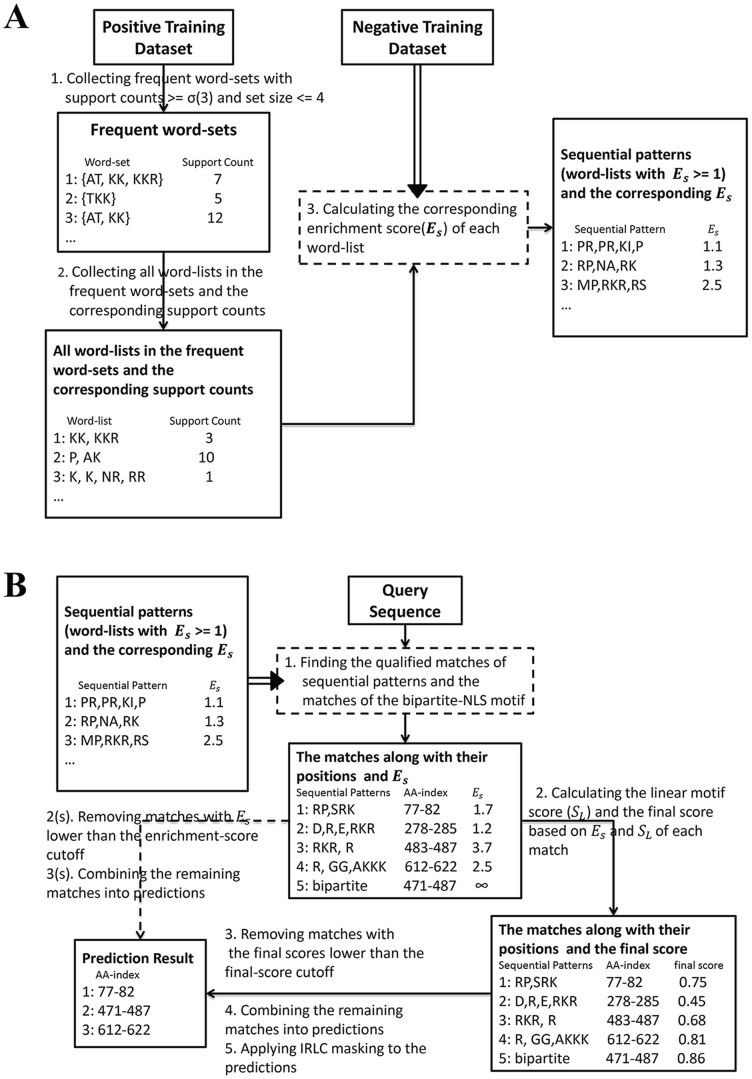
The flow charts of predicting NLS. (a) The flow chart of mining the sequential patterns. (b) The flow chart of predicting NLS on a query sequence; the dashed line corresponds to the sequence-based predictor, and the other branch using linear motif scoring refers to the integrated prediction algorithm.

Figure 1(a) shows the flow chart of NLS sequential-pattern mining on a training dataset. We first define a segment of amino acids as a word, and a set of words in sequential order as a word-list; the NLS sequential patterns are thus defined as word-lists over-expressed in a set of NLSs (positive training dataset) against a set of peptides not overlapped with any NLS (negative training dataset). The number of different word-lists within the positive training dataset is too large while many of them are redundant; therefore, we limit the search space of word-lists as frequent word-sets within the positive training dataset, which can effectively reduce the search space and maintain the diversity of word-lists; the frequent word-set is defined as a word-set with support count no less than 3 within the positive training dataset and set size not larger than 4. For example, if there are 12 NLSs in the positive training dataset containing the word-set {AT, KK}, the word-set {AT, KK} is a frequent word-set since its support count is 12 and the set size is 2. We apply the frequent item-set mining algorithm [27] to collect all the frequent word-sets within the positive training dataset in step 1; the word-lists are obtained by permuting each of the frequent word-sets, and the corresponding support counts in the positive training dataset are then collected in step 2; in step 3, all the word-lists are scored according to their corresponding occurrences in the positive and negative training datasets to measure their enrichment. The enrichment score is defined as follows:
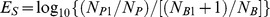
where E_S_ is the enrichment score, N_P_ is the number of NLSs in the positive training dataset while N_P1_ is the number of NLSs in the positive training dataset containing the word-list, and N_B_ is the number of peptides of length 40 that are not overlapped with NLS in the negative training dataset while N_B1_ is the number of peptides in the negative training dataset that contain the word-list; N_P_ is 108 and N_B_ is 26772 according to our training dataset. The enrichment score E_S_ is essentially a measure of over-representation for the word-lists in the training NLSs relative to the non-NLS peptides. The word-lists with E_S_ not lower than a default threshold 1.0 are collected as the sequential patterns, which will then be used to detect segments which are likely to be (parts of) a NLS in a query sequence.

#### Detecting potential NLS segments by using the NLS sequential patterns

The process to detect potential NLS segments by using the collected NLS sequential patterns is illustrated in Figure 1(b). First, the collected sequential patterns are used to find qualified matches in the query sequence, which are defined as the matches of the sequential patterns in the query sequence with each gap between the words no longer than two amino acids. The reason to limit the length of the gaps is to maintain the statistical significance of the sequential-pattern matches since it is much more likely to have words in a specific order by chance when long gaps are allowed. These qualified matches are recognized as potential NLS segments in our algorithm, of which E_S_ is a measure of the significance of these potential NLS segments to be true NLS. In Figure 1(b), the dashed line corresponds to our sequence-based predictor, and the other branch using linear motif scoring refers to our integrated prediction algorithm.

### Incorporation of bipartite-NLS motifs

Our SeqNLS algorithm does not make any assumptions over the type of the predicted NLSs. However, to improve the prediction performance, a bipartite-NLS motif is incorporated in SeqNLS to increase the sensitivity of detecting bipartite NLSs. Bipartite NLSs are a class of classical NLS usually composed of two clusters of basic amino acids separated by a gap of 10–12 residues [28], [29] while longer gaps are also possible [30]. Bipartite NLSs are very common as it was approximated that 25.8% of proteins localized to the nucleus contain putative bipartite NLSs [7]. Several consensus patterns of bipartite NLSs have been defined such as (K/R)(K/R)X10–12(K/R)3/5 [31], KRX10–12KRRK [32], and KRX10–12K(K/R)(K/R) or KRX10–12K(K/R)X(K/R) [25], where (K/R)3/5 represents any 5 consecutive amino acids having at least three of either lysine or arginine. Since bipartite NLSs have long gaps between the two words, they may not be detected by our sequential-pattern mining method. Therefore, we included a bipartite-NLS motif (K/R)(K/R)X10(K/R)3/5, which is also used to predict bipartite NLS in PSORTII, to complement the motifs mined from the training NLSs. As shown in Figure 1(b), when detecting potential NLS segments, our algorithm also collects the matches of the bipartite-NLS motif in addition to the qualified matches of the sequential patterns. The matches of the bipartite-NLS motif were found usually more reliable than the matches of sequential patterns according to our experiment result. Therefore the enrichment score of the matches of the bipartite-NLS motif is set as an arbitrarily large value which will never be lower than the enrichment-score cutoff as defined in the next paragraph.

### Predicting NLS based on sequence features only: sequence-based predictor

Given a query sequence, the extracted sequential patterns along with the bipartite-NLS motif are used to scan it for matches. Those matches with E_S_ score lower than a pre-defined cutoff will be removed (the matches of the bipartite-NLS motif will never be removed). The remaining matches will then be combined using a merging procedure: every two overlapped matches are merged into one match of which the boundaries are defined as the union of the overlapped matches. The merging process will continue until all the matches are not overlapped. The resulting matches will be the output of the sequence-based NLS predictor.

### Linear motif scoring

To further improve the performance of NLS prediction, we developed a linear motif-scoring scheme to remove the false positives of the matches as obtained above based on the linear motif attributes. NLSs are one kind of linear motifs, which are found to predominantly occur in disordered regions [23], [33]. One possible reason is that disordered regions can provide linear motifs unstructured interfaces to adapt to the interacting partner with higher flexibility. In addition, evolutionary plasticity inherent to disordered regions increases the likelihood of evolving linear motifs [23]. To exploit this preference of linear motifs, we used PrDOS [34], one of the best-performing disorder predictors according to CASP9 [35], to predict disorder scores for all residues in the query sequence. Given a predicted amino acid segment, the median disorder score of residues within the segment is defined as the disorder score of the predicted peptide.

Another factor to estimate the likelihood of linear motifs is residue accessibility, which is required for linear motifs to function; deeply buried residues are less likely to interact with the partner proteins [36]. In our experiments, NetSurfP [37] was used as the residue-accessibility predictor, and the relative surface area (RSA) was used as the measure of residue accessibility. Given a predicted amino acid segment, the median RSA score of residues within the segment is defined as its RSA score.

Our linear motif-scoring scheme is implemented by estimating the probability of being NLS for a given peptide. We call this probability as the linear motif score (S_L_). It is calculated by building a Support Vector Machine (SVM) classifier based on the aforementioned linear motif attributes, whose output is the probability of an input amino acid segment belonging to the NLS class. We collected 90 NLSs and 90 non-NLS peptides (mentioned in the section “Training and Testing dataset”) as the positive and negative training datasets for the SVM. The linear motif attributes including the PrDOS disorder score and the NetSurfP RSA score were used as the features. The SVM classifier was trained using the LIBSVM package [38] with the radial basis function as the kernel, and the probability of being NLS for a given input peptide was obtained by calculating the probability estimation of LIBSVM.

### Predicting NLS based on sequence and linear motif scoring: SeqNLS, the integrated predictor

Our SeqNLS algorithm works by sequential-pattern mining and matching plus linear motif scoring. First, it collects the matches of the sequential patterns and the bipartite-NLS motif in the query sequence. Next, all the matches of the sequential patterns and the bipartite-NLS motif will be estimated the probability of being NLS by linear motif scoring. The respective linear motif score will then be combined with the corresponding enrichment score to generate the final score. The matches whose final scores lower than a predefined cutoff will be removed.

To combine the enrichment score and the linear motif score, we defined the normalized enrichment score which has the same scale as the linear motif score (between 0 and 1). According to our experiment result, we found that when the enrichment-score cutoff is over a certain threshold E_K_, the prediction precision cannot be improved by further increasing the cutoff. The normalized enrichment score is thus defined according to the following formula:
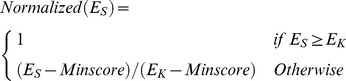
where Normalized(E_S_) represents the normalized enrichment score, and Minscore represents the minimal possible score of E_S_, which is 1 according to our setting since only sequential patterns with E_S_ greater or equal to 1 are collected. The final score will then be calculated according to the following formula:
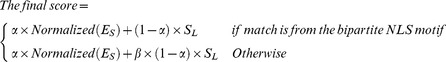



It should be noted that the SVM model of calculating S_L_ is trained to discriminate between NLSs and peptides not overlapped with NLS; however, those true positive matches, which are matches overlapped with NLS according to our definition, do not always have accurate NLS boundaries; the more accurate the NLS boundaries of the true positive matches are, the more reliable their S_L_ will be. In the formula, S_L_ of the sequential-pattern matches is multiplied by a weighting factor *β* (smaller than 1) because we found that the true positive matches of the bipartite-NLS motif generally have more accurate NLS-boundaries in terms of residue-level accuracy. In our study the optimal *α* and *β* are set as 0.8 and 0.6 respectively.

### IRLC-masking

Due to the short and degenerate nature of linear motifs, the evolutionary conservation of linear motifs cannot be well represented by simple sequence-alignment models. Davey et al [39] proposed the relatively local conservation (RLC) score, which measures the conservation of residues relative to their neighboring regions. They applied RLC masking to remove residues unlikely to be functional residues within linear motifs, based on the rationale that functional residues should be more conserved than the neighboring regions. While RLC masking has been used to remove false positive matches of known linear motifs [39], it is not an appropriate method to remove false positive NLS predictions due to the fact that those true positive NLS predictions, unlike the true positive matches of other linear motifs, do not always have accurate NLS boundaries and may cover non-functional residues while wildcard positions are not known. Therefore, we proposed the inverse relative local conservation (IRLC) scheme to remove false positive NLS predictions based on the following rationale: since linear motifs are more conserved than their flanking residues, the chance to have a flanking residue which is much more conserved than the residues within the linear motif should be very small.

To evaluate IRLC, we first define M as the mean conservation score of N residues within a predicted NLS:
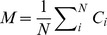
where C_i_ is the conservation score representing the degree of conservation of a residue in position i of the predicted NLS; C_i_ can be calculated by any suitable scoring metric, while in our experiment, position specific scoring matrix (PSSM) was used to evaluate residue conservation; the conservation score of a residue in the position i' of a sequence was obtained from the corresponding column of the residue in the i'-th row of the PSSM of the sequence. The PSSM of each query sequence was generated by three iterations of PSI-BLAST [40] searches against NCBI non-redundant database with the BLOSUM62 substitution matrix and E-value threshold of 0.001. Second, we define IRLC_j_ as the IRLC score for a flanking residue j:
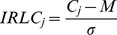
where the flanking residues are defined as the residues within 5 amino acids away from the predicted NLS, and σ represents the standard deviation of the conservation scores of all the residues in the sequence. The IRLC score for a NLS prediction can thus be defined as:
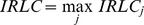



A NLS prediction will be determined as a false positive prediction if its IRLC score is higher than some threshold value T. The rationale is that if there is any residue in the flanking region that is much more conserved than the average conservation score of the region of interest, it is less likely that the region of interest represents a functional NLS since it contradicts the property of relative local conservation of linear motifs.

### Performance evaluation

To evaluate NLS prediction performance, a NLS prediction is considered a hit if the prediction is overlapped with at least one annotated NLS in the testing dataset otherwise it is labeled as a miss. Three performance metrics are defined to evaluate NLS prediction performance as follows:
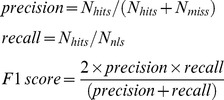
where N_hits_ is the number of hits, N_miss_ is the number of misses, and N_nls_ is the number of NLSs in the testing dataset. In addition, we introduced the amino acid level performance coefficient [41] (aPC) to evaluate the amino acid-level accuracy of a predicted peptide overlapped with NLS. The aPC is defined as follows:
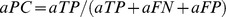
where aTP represents the number of amino acids of a predicted NLS that are overlapped with the true NLS; aFP represents the number of amino acids of a predicted NLS that are not overlapped with the true NLS; aFN represents the number of amino acids of the true NLS that are not overlapped with the predicted NLS. In our evaluation, the mean aPC of all the true positive predictions (Mean aPC) is defined to evaluate the amino acid level accuracy of a predictor.

## Results and Discussion

### Performance of the sequence-based NLS predictor

**Figure 2:**
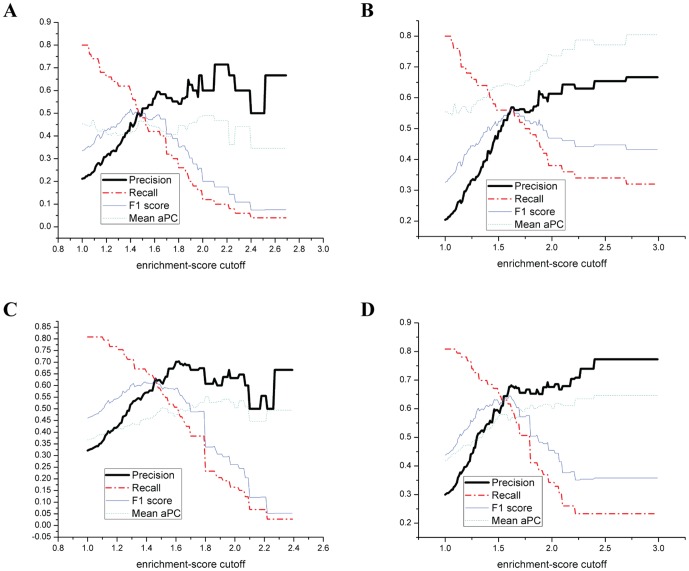
The prediction performance of the sequence-based predictor. (a) The Yeast dataset; the bipartite-NLS motif is not incorporated. (b) The Yeast dataset; the bipartite-NLS motif is incorporated. (c) The Hybrid dataset; the bipartite-NLS motif is not incorporated. (d) The Hybrid dataset; the bipartite-NLS motif is incorporated.

We applied the sequence-based predictor to the Yeast and Hybrid datasets, and the result is shown in Figure 2. It shows that when the enrichment-score cutoff is set higher, the precision of the predictor increases. This is because the matches of the sequential patterns with the higher enrichment score are more significant and thus are more likely to be part of NLS. However, in Figure 2(a) and 2(c), it can be shown that for both the Yeast dataset and the Hybrid dataset, when the enrichment-score cutoff is higher than 1.62, no obvious precision improvement can be obtained by further raising the cutoff. We thus set E_K_ as 1.62 in our experiment. In the meantime, recall decreases with the increase of the enrichment-score cutoff. This is because fewer matches can meet the higher enrichment-score cutoff, and thus fewer annotated NLSs can be covered by the matches. The performance of the predictor incorporated with the bipartite-NLS motif is shown in Figure 2(b) and 2(d). It was found that precision can be further improved by setting a higher enrichment-score cutoff even when the cutoff is higher than 1.62 (E_K_). It implies that the bipartite-NLS motif is a more reliable NLS pattern than the mined sequential patterns; by setting the higher enrichment-score cutoff, the proportion of the sequential-pattern matches will become smaller, and the matches of the bipartite-NLS motif will dominate prediction performance when the enrichment-score cutoff is much higher than E_K_.

To evaluate the performance of the bipartite-NLS motif in NLS prediction, we evaluated the performance of the sequence-based predictor using only the bipartite-NLS motif in Table 1 and Table 2 (last column). It was shown that the predictor using only the bipartite-NLS motif has high precision on the both datasets: 0.667 on the Yeast dataset and 0.77 on the Hybrid dataset. It also has very high residue-level accuracy: the Mean aPC is 0.805 and 0.645 on the Yeast dataset and the Hybrid dataset respectively while the Mean aPC of most other NLS predictors is around 0.4 to 0.5. The high precision of the bipartite-NLS motif based predictor is probably due to the high specificity of the bipartite-NLS motif pattern. However, the recall of this method is only 0.32 and 0.233 respectively on the Yeast dataset and the Hybrid dataset.

**Table 1:** The prediction performance of the sequence-based predictor with different enrichment-score cutoffs with and without incorporating the bipartite-NLS motif on the Yeast dataset.

Enrichment- score cutoff		1.0	1.2	1.4	1.6	1.8	2.0	2.2	2.3	Bi-Partite*
Precision		0.212	0.311	0.458	0.564	0.555	0.6	0.714	0.6	0.667
	+BiPartite	0.204	0.303	0.427	0.547	0.558	0.613	0.643	0.63	
Recall		0.8	0.66	0.6	0.42	0.26	0.12	0.1	0.06	0.32
	+BiPartite	0.8	0.68	0.62	0.56	0.48	0.38	0.36	0.34	
F1 score		0.335	0.423	0.519	0.482	0.351	0.2	0.175	0.109	0.432
	+BiPartite	0.325	0.418	0.505	0.554	0.516	0.469	0.462	0.442	
Mean aPC		0.453	0.413	0.412	0.443	0.446	0.49	0.467	0.442	0.805
	+BiPartite	0.554	0.563	0.607	0.645	0.686	0.736	0.757	0.788	

* Predictions with only the bipartite-NLS motif: (K/R)(K/R)X_10_(K/R)_3/5_.

Mean aPC: the mean aPC of all the true positive predictions as defined in the text.

**Table 2:** The prediction performance of the sequence-based predictor with different enrichment-score cutoffs with and without incorporating the bipartite-NLS motif on the Hybrid dataset.

Enrichment- score cutoff		1.0	1.2	1.4	1.6	1.8	2.0	2.2	2.3	Bi-Partite*
Precision		0.322	0.421	0.57	0.702	0.607	0.632	0.556	0.667	0.77
	+BiPartite	0.3	0.399	0.546	0.677	0.652	0.676	0.704	0.739	
Recall		0.808	0.767	0.658	0.507	0.233	0.164	0.068	0.027	0.233
	+BiPartite	0.808	0.781	0.685	0.616	0.411	0.342	0.26	0.233	
F1 score		0.46	0.544	0.611	0.589	0.337	0.261	0.122	0.053	0.358
	+BiPartite	0.438	0.528	0.608	0.645	0.504	0.455	0.38	0.354	
Mean aPC		0.367	0.416	0.46	0.475	0.552	0.504	0.447	0.494	0.646
	+BiPartite	0.418	0.473	0.534	0.56	0.612	0.601	0.61	0.634	

* Predictions with only the bipartite-NLS motif: (K/R)(K/R)X_10_(K/R)_3/5_.

Mean aPC: the mean aPC of all the true positive predictions as defined in the text.

To evaluate if the bipartite-NLS motif can help to improve the sequence-based predictor, the prediction performance of the sequence-based predictor with or without incorporating the bipartite-NLS motif is shown in Table 1 and Table 2. It is shown that recall can be improved on both the Yeast and Hybrid datasets after incorporating the bipartite-NLS motif. Improvement on recall depends on the enrichment-score cutoff: when the enrichment-score cutoff is lower, more bipartite NLSs in the testing datasets could be partially covered (overlapped) by the sequential-pattern matches, and thus improvement on recall is smaller. Alternatively, when the cutoff score is higher than 1.6, the incorporation of the bipartite-NLS motif significantly improves recall. Besides, the Mean aPC can be significantly improved by incorporating the bipartite-NLS motif: when the enrichment-score cutoff is set as 1.6, the Mean aPC can be improved from 0.443 to 0.645 on the Yeast dataset and from 0.475 to 0.56 on the Hybrid dataset. Improvement on the Mean aPC also depends on the enrichment-score cutoff: when the enrichment-score cutoff is lower, more bipartite NLSs in the testing dataset are likely to be overlapped with the matches and thus improvement on the Mean aPC by incorporating the bipartite-NLS motif is less obvious. In addition, improvement on both recall and the Mean aPC by incorporating the bipartite-NLS motif also depends on the ratio of bipartite NLSs in the testing datasets, which explains why the improvement on the Yeast dataset is greater than that of the Hybrid dataset.

From Table 1 and Table 2, we also found that when the enrichment-score cutoff is set as 1.0, 80% of the NLSs can be covered by our sequential-pattern matches for both the Yeast and Hybrid datasets. This indicates that our sequential patterns with the enrichment score higher than 1.0 cover 80% of NLSs, which can be used in searching potential NLSs extensively.

### Linear motif attributes of NLS

**Figure 3:**
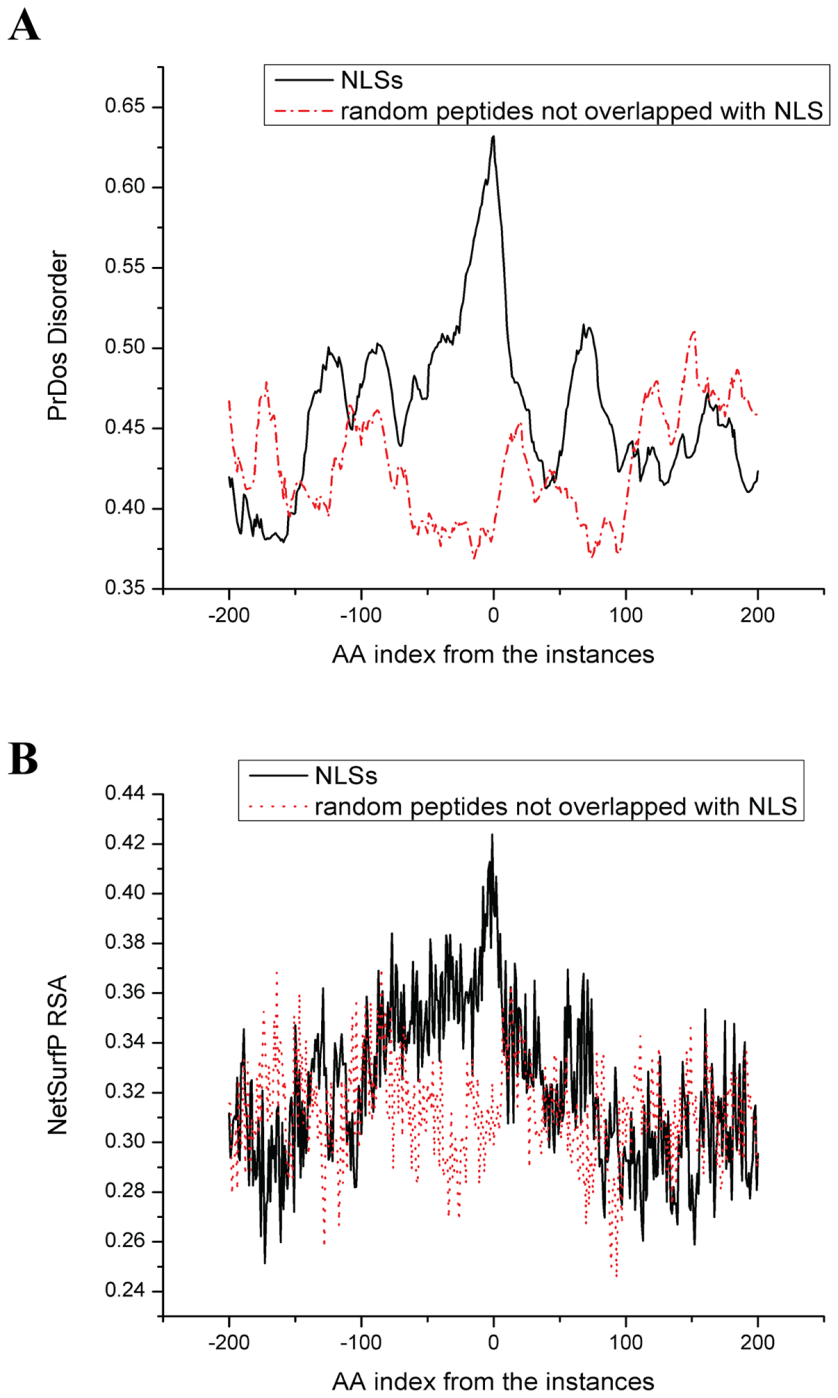
The linear motif attributes of NLSs. (a) PrDOS disorder scores of the 200 residues either side of the annotated NLSs and random peptides not overlapped with NLS. (b) NetSurfP RSA values of the 200 residues either side of the annotated NLSs and random peptides not overlapped with NLS. The index 0 represents the residue at the boundary of the left or right side of the NLS (or peptide).

Here we evaluate the discriminative capacity of linear motif attributes for NLS identification. Figure 3(a) shows the disorder propensity of NLSs: the mean PrDOS disorder score of the 90 training NLSs is 0.632 while the mean PrDOS disorder score of the 90 peptides not overlapped with NLS is 0.386. The disorder propensity of NLSs is clearly shown by the peak at index 0, while no such preference exists for the peptides not overlapped with NLS.

Figure 3(b) shows the RSA propensity of NLSs: the mean NetSurfP RSA score of the 90 training NLSs is 0.393, while the mean NetSurfP RSA score of the 90 peptides not overlapped with NLS is 0.299. The preference of NLSs for higher RSA is also observed by the peak at index 0, while no such preference exists for the peptides not overlapped with NLS. Compared to the disorder propensity, the RSA propensity of NLSs is less significant since the difference of the mean attribute value between NLSs and peptides not overlapped with NLS is 0.094 for NetSurfP RSA, while it is 0.246 for PrDOS disorder (the PrDOS disorder score and the NetSurfP RSA score both have the same scale 0–1).

**Figure 4:**
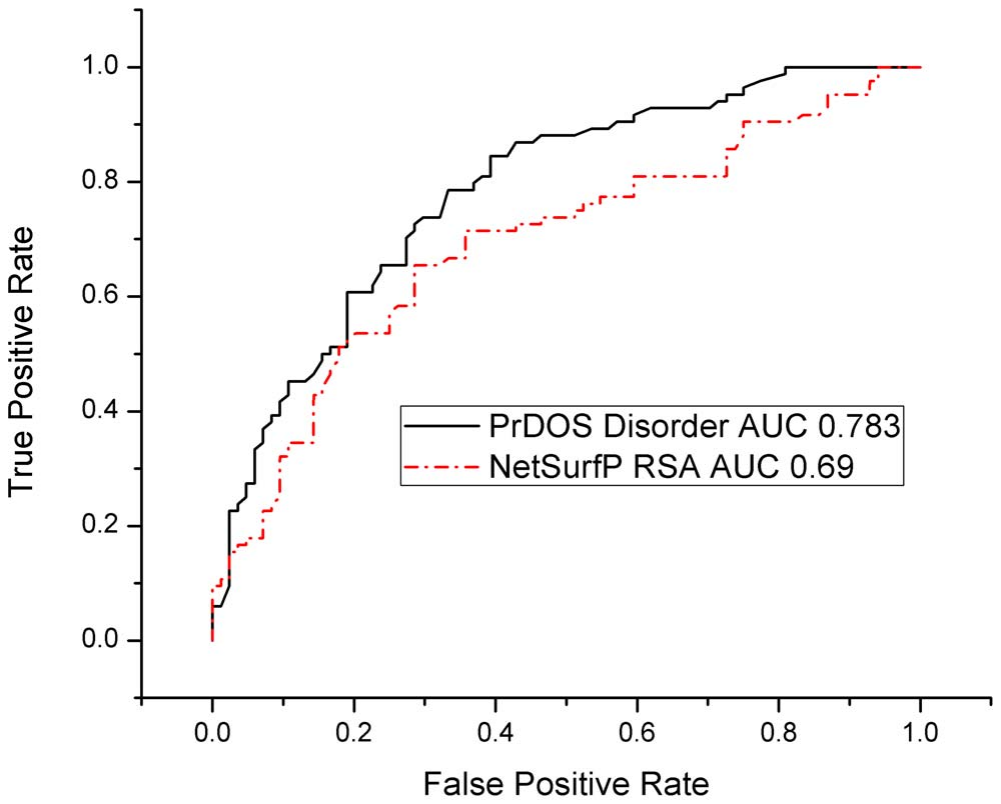
ROC curves for the PrDOS disorder feature and NetSurfP RSA feature.

To further investigate the discriminative capacity of these attributes, we first used each of the attributes to build a single-feature binary classifier in which the prediction is based on the cutoff of the attribute value. The ROC curves of the binary classifiers are plotted in Figure 4. As shown in the figure, the AUC values for the PrDOS disorder score and the NetSurfP RSA score are 0.783 and 0.69 respectively. This suggests that PrDOS disorder and NetSurf RSA are both useful features to discriminate between NLS and non-NLS peptides. We further used each of the attributes to build a single-feature SVM classifier. The LIBSVM package with the radial basis function kernel was used to run a 5-fold cross-validation on the 90 NLSs and 90 non-NLS peptides in the training dataset. We found that when the PrDOS disorder score of the peptide was used as the single feature, it achieved a 5-fold cross-validation accuracy of 70.83% on discriminating NLS and non-NLS peptides; while using the NetSurfP RSA score of the peptide as the single feature, it achieved 64.88% accuracy; when both the PrDOS disorder score and the NetSurfP RSA score of the peptide were used as the features, the accuracy was 70.24%, which was not higher than that of using the PrDOS disorder score alone. This indicates that although the NetSurfP RSA score is also a discriminative attribute, it is redundant if the PrDOS disorder score is used. Therefore, in our following experiments only the PrDOS disorder score is used in the linear motif scoring to estimate the probability of being NLS.

### Performance of the integrated predictor: SeqNLS

**Figure 5:**
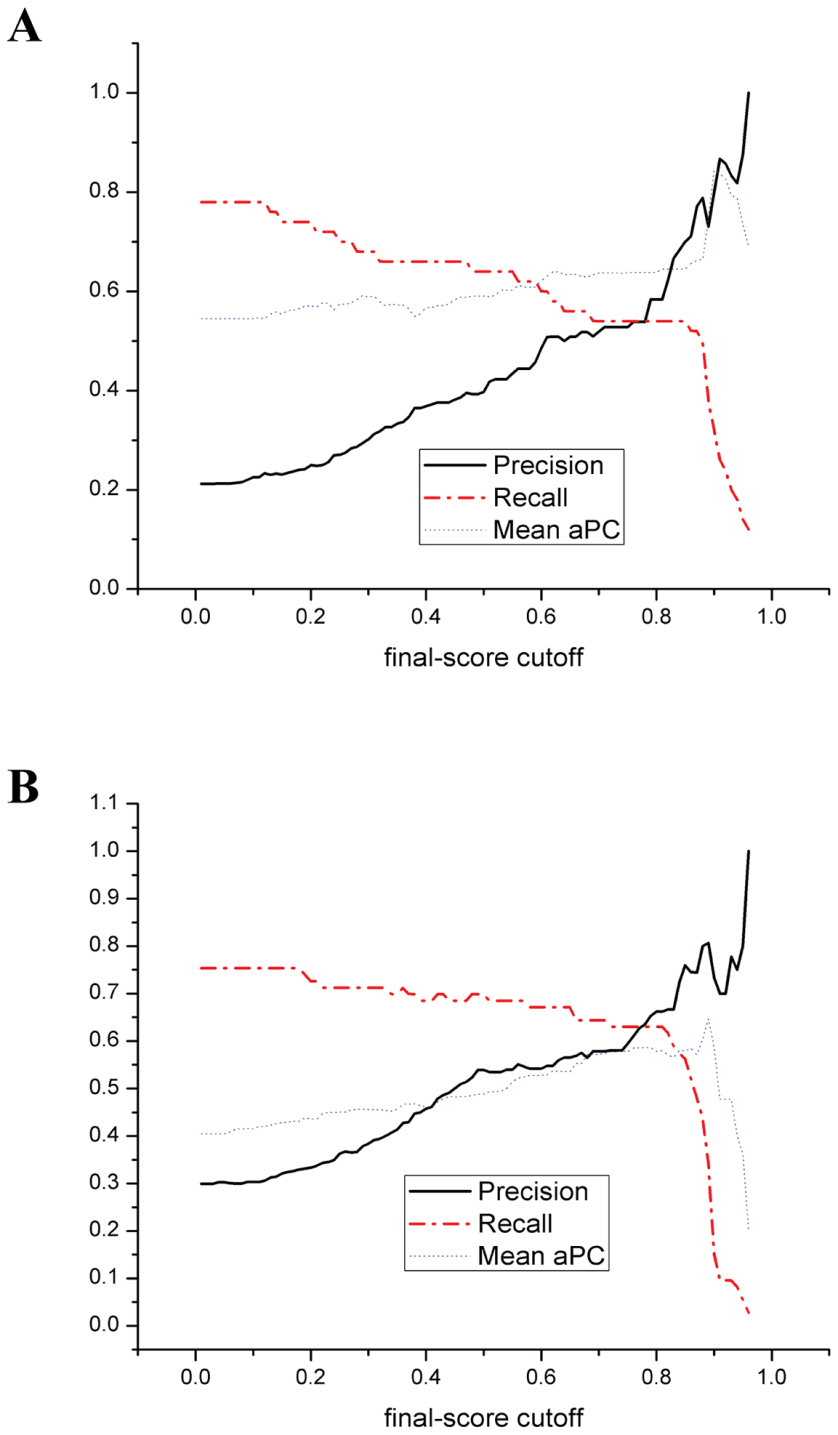
The prediction performance of the integrated predictor (a) The Yeast dataset (b) The Hybrid dataset. IRLC masking is applied in both (a) and (b).

Figure 5 shows the prediction performance of SeqNLS on the Yeast and Hybrid datasets. The algorithm attains a precision and recall of 0.7 and 0.5 or higher when the final-score cutoff is set as 0.85. By tuning the final-score cutoff, the algorithm can attain different precision and recall rates with the higher final-score cutoff leading to higher precision and lower recall. The higher final-score cutoff also leads to the higher Mean aPC, which indicates that matches with the higher final scores generally are less likely to cover non-NLS amino acids. As indicated previously, the highest precisions of the sequence-based predictor are 0.667 and 0.77 respectively on the Yeast dataset and the Hybrid dataset by maximizing the enrichment-score cutoff. For the integrated predictor, precision can be further improved to around 0.75 to 0.8 on both the Yeast dataset and the Hybrid dataset while a higher recall is maintained. This implies that the proposed linear motif scoring and IRLC-masking improve the prediction.

Figure 5 also shows that recall starts dropping dramatically when the final-score cutoff exceeds certain value over 0.8 on the both Yeast and Hybrid datasets. This is because matches with the enrichment scores higher than E_K_ certainly have the final scores at least 0.8 according to the formula of calculating the final score. These matches cover 56% and 60.3% of NLSs (see Figure 2(b) and Figure 2(d)) in the Yeast and Hybrid datasets. Therefore, recall won't drop dramatically when the final-score cutoff is lower than 0.8. When the final-score cutoff is set higher than 0.8, matches with the low enrichment scores are removed since the weight of the enrichment score is much higher than that of the linear motif score (0.8 vs. 0.2); with the increase of the cutoff afterward, matches with high enrichment scores but low linear motif scores will start being removed, and eventually only matches with high enrichment scores and high linear motif scores are left. The result shows that for both the Yeast and Hybrid datasets, the precision of the integrated predictor can still be improved by increasing the final-score cutoff even when the final-score cutoff is already higher than 0.8. This indicates that matches with low linear motif scores are less likely to be (part of) NLS despite their high enrichment scores. Therefore, the enrichment score and the linear motif score are highly complementary in discerning NLS.

### Effect of IRLC-masking

**Figure 6:**
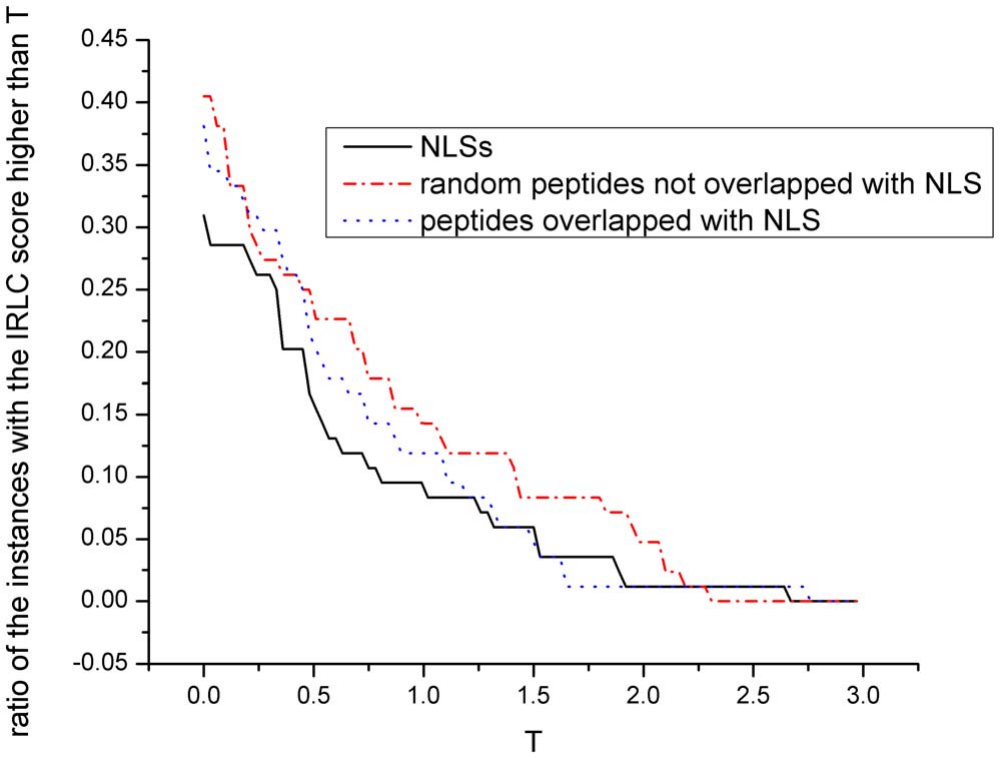
The effect of IRLC-masking. Peptides overlapped with NLS are obtained by adjusting boundaries of the NLSs to upstream or downstream proteins randomly in the parent by 1/3 length of the corresponding NLSs.

Figure 6 shows the ratio of three types of peptides in our training dataset with the IRLC scores higher than a threshold value T. It can be observed that the ratio of NLSs with the IRLC score higher than T is smaller than that of random peptides that are not overlapped with NLS. This result corresponds to our IRLC hypothesis that the chance is relatively low to find a residue in the flanking region of a NLS that is much more conserved; in other words, NLSs indeed tend to have higher relative local conservation. The similar trend can be observed for peptides partially overlapped with NLSs, which mimics true positive NLS predictions. This implies that IRLC-masking may be effective in masking out false positive NLS predictions with a smaller chance of masking out true positive NLS predictions. Figure 6 also shows that when T is higher than 1.7, both the ratios of NLSs and peptides overlapped with NLS with the IRLC score higher than T are close to 0. To avoid masking out any true positive predictions, the IRLC-masking cutoff is set as 1.7 throughout our experiment.

Table 3 and Table 4 describe the prediction performance of the integrated predictor with or without IRLC-masking on the Yeast and Hybrid datasets respectively. It shows that IRLC-masking improves the precision of the integrated predictor on the Yeast dataset while it is not effective on the Hybrid dataset. This is because the effect of IRLC-masking depends on where false positive predictions are distributed: if no false positive predictions are located in the regions of the sequence that contradict the property of relative local conservation (RLC), the precision cannot be improved. This can also explain why precision is not improved by applying IRLC-masking on the Yeast dataset when the final-score cutoff is higher than or equal to 0.9. In addition, it shows that for the both datasets after IRLC-masking is applied, recall decreases slightly when the final-score cutoff is lower than 0.8 while it remains the same when the final-score cutoff is higher than 0.8. This is because the true positive predictions coming from matches with the lower final scores generally have less accurate boundaries, which lead to more true positive predictions being masked out by IRLC-masking.

**Table 3:** The prediction performance of the integrated predictor with different final-score cutoffs with and without IRLC masking on the Yeast dataset.

Final-score cutoff		0.6	0.65	0.7	0.75	0.8	0.85	0.9	0.95
Precision		0.438	0.462	0.483	0.492	0.537	0.651	0.8	0.875
	IRLC	0.484	0.509	0.518	0.528	0.583	0.7	0.8	0.875
Recall		0.62	0.58	0.56	0.56	0.56	0.54	0.32	0.14
	IRLC	0.6	0.56	0.54	0.54	0.54	0.54	0.32	0.14
F1 score		0.514	0.514	0.519	0.524	0.548	0.59	0.457	0.241
	IRLC	0.536	0.533	0.529	0.534	0.561	0.61	0.457	0.241
Mean aPC		0.623	0.635	0.638	0.639	0.639	0.644	0.844	0.734
	IRLC	0.621	0.634	0.637	0.638	0.638	0.644	0.844	0.734

Mean aPC: the mean aPC of all the true positive predictions as defined in the text.

**Table 4:** The prediction performance of the integrated predictor with different final-score cutoffs with and without IRLC masking on the Hybrid dataset.

Final-score cutoff		0.6	0.65	0.7	0.75	0.8	0.85	0.9	0.95
Precision		0.541	0.564	0.583	0.6	0.662	0.759	0.733	0.8
	IRLC	0.542	0.565	0.578	0.595	0.662	0.759	0.733	0.8
Recall		0.685	0.685	0.658	0.644	0.63	0.562	0.151	0.055
	IRLC	0.671	0.671	0.644	0.63	0.63	0.562	0.151	0.055
F1 score		0.604	0.619	0.618	0.621	0.646	0.646	0.25	0.103
	IRLC	0.6	0.614	0.609	0.612	0.646	0.646	0.25	0.103
Mean aPC		0.532	0.539	0.575	0.58	0.578	0.579	0.587	0.361
	IRLC	0.528	0.535	0.572	0.577	0.578	0.579	0.587	0.361

Mean aPC: the mean aPC of all the true positive predictions as defined in the text.

### Comparison of SeqNLS with state-of-the-art NLS prediction algorithms

Here we compare the prediction performance of SeqNLS with those of state-of-the-art NLS prediction algorithms. Considering that some of the compared NLS predictors may generate overlapped NLS predictions, for all the compared NLS predictors, if two NLS predictions are overlapped, they will be merged into one prediction before evaluation. Table 5 and Table 6 show the prediction performance of different NLS prediction methods on the Yeast and Hybrid datasets respectively. We can see that PSORTII has the highest recall on the both datasets while its precision is the lowest among all the methods. This indicates that many NLSs and non-NLS peptides can be covered by the NLS patterns used in PSORTII. An interesting observation is that PSORTII has the highest Mean aPC. We investigated the individual patterns used in PSORTII and found that its high Mean aPC is attributed to the predictions of the bipartite-NLS pattern (K/R)(K/R)X10(K/R)3/5. PredictNLS only generated a small number of predictions as shown by its low coverage in terms of recall. It was found that both NLStradamus and cNLS mapper have very high precision on the Yeast dataset. This is partially due to that our Yeast dataset is included in the training data of the NLStradamus prediction server and the activity profiles built in cNLS mapper are optimized for yeast. For the Hybrid dataset, both NLStradamus and cNLS mapper exhibit lower precision since this dataset is not overlapped with the Yeast dataset and includes many different species in addition to the yeast species, of which the collected NLSs are from literature after 2010. The NucImport algorithm has a very poor Mean aPC score because its NLS predictions have uniform length of 20 amino acids. Another limitation of NucImport is that it can predict only one NLS per sequence while in the testing datasets some NLSs occur within the same parent proteins.

**Table 5:** The prediction performance of different NLS predictors on the Yeast dataset.

Yeast Dataset	PSORT II	PredictNLS	NLStradamus	cNLS Mapper	NucImport	1Sequence-based predictor	2Integrated predictor
Precision	0.455	0.462	0.864	0.8	0.526	0.569	0.7
Recall	0.66	0.12	0.36	0.46	0.4	0.56	0.54
F1 score	0.538	0.19	0.508	0.584	0.455	0.564	0.61
Mean aPC	0.696	0.411	0.473	0.437	0.414	0.641	0.644

1 Sequence-based predictor with the enrichment-score cutoff set as 1.62 (E_K_).

2 Integrated predictor with the final-score cutoff set as 0.85 with IRLC masking.

Mean aPC: the mean aPC of all the true positive predictions as defined in the text.

**Table 6:** The prediction performance of different NLS predictors on the Hybrid dataset.

Hybrid Dataset	PSORT II	PredictNLS	NLStradamus	cNLS Mapper	NucImport	1Sequence-based predictor	2Integrated predictor
Precision	0.617	0.857	0.714	0.696	0.632	0.682	0.759
Recall	0.671	0.151	0.329	0.425	0.329	0.603	0.562
F1 score	0.643	0.256	0.45	0.527	0.432	0.64	0.646
Mean aPC	0.657	0.455	0.56	0.446	0.358	0.57	0.579

1 Sequence-based predictor with the enrichment-score cutoff set as 1.62 (E_K_).

2 Integrated predictor with the final-score cutoff set as 0.85 with IRLC masking.

Mean aPC: the mean aPC of all the true positive predictions as defined in the text.

As shown in Table 5 and Table 6, our sequence-based predictor with the enrichment-score cutoff set as 1.62 (E_K_) has comparable or better prediction performance than other NLS prediction methods: it achieved a recall rate of 0.56 and 0.603 on the Yeast dataset and the Hybrid dataset respectively, which is only second to PSORTII. However, its precision is better than PSORTII on both datasets. The integrated predictor shows better precision than the sequence-based predictor since it incorporates linear motif attributes. When the final-score cutoff is set as 0.85, the integrated predictor achieved a precision of 0.7 and 0.759 on the Yeast and the Hybrid datasets respectively while its recall is 0.54 on the Yeast dataset and 0.562 on the Hybrid dataset. That is, over 50% of the NLSs can be covered. The reason that the integrated predictor can achieve high precision while maintaining high recall is that the algorithm can extensively detect potential NLSs by using the sequential-pattern mining method while exploiting linear motif scoring, which is not used by other NLS prediction methods. As for residue-level accuracy, both the sequence-based predictor and the integrated predictor achieve the higher Mean aPC compared to most other NLS prediction methods because of its incorporation of the bipartite-NLS motif. It is interesting to note that another example of achieving better prediction performance by integrating sequence features and predicted disorder is NESsential [42], which is a computational method designed to predict nuclear export signals (NESs).

## Conclusion

In this study, we propose SeqNLS, a novel method for nuclear localization signal prediction based on frequent pattern mining and linear motif scoring. Various attributes of NLS including the sequential-pattern enrichment, predicted disorder, and local conservation are investigated based on the two well-curated datasets, which demonstrates their discriminative capacity for identifying NLSs. Our experimental results indicate that sequence features in terms of sequential patterns and linear motif features are highly complementary for NLS prediction. Compared to other state-of-the-art NLS prediction methods, SeqNLS achieves better overall prediction performance. For the Yeast and Hybrid datasets, SeqNLS attains a F1 score of 0.61 and 0.646 respectively compared to 0.538 and 0.643 of PSORT-II.

## Supporting Information

Table S1 **The Yeast NLS dataset.**
(DOCX)

Table S2 **The Hybrid NLS dataset.**
(DOCX)
