# Supplementary material for: SeqNLS: Nuclear Localization Signal Prediction Based on Frequent Pattern Mining and Linear Motif Scoring
Source: PLoS One. 2013 Oct 29;8(10):e76864. doi: 10.1371/journal.pone.0076864 (PMC3812174; doi:10.1371/journal.pone.0076864)
Supplement: Table S1 — The Yeast NLS dataset. (DOCX) [file pone.0076864.s001.docx]

Table S1. The Yeast NLS dataset

| UniProtKB ID | Start | Stop | Referenced PMID |
| --- | --- | --- | --- |
| YBR009C | 4 | 21 | 11694505 |
| YBR010W | 10 | 28 | 11694505 |
| YDL007W | 11 | 15 | 15210724 |
| YDL007W | 33 | 37 | 15210724 |
| YDR103W | 49 | 66 | 10481914 |
| YDR146C | 636 | 655 | 7615496 |
| YDR208W | 347 | 364 | 12912920 |
| YEL009C | 231 | 246 | 12455686 |
| YER040W | 388 | 394 | 12624103 |
| YGL071W | 202 | 207 | 14523005 |
| YGL071W | 352 | 355 | 14523005 |
| YGL071W | 332 | 335 | 14523005 |
| YGL097W | 3 | 23 | 18485366 |
| YHR079C | 645 | 657 | 17035634 |
| YIL075C | 811 | 832 | 15210724 |
| YIL150C | 512 | 527 | 13680157 |
| YIL150C | 435 | 451 | 13680157 |
| YJL194W | 27 | 33 | 18485366 |
| YLR103C | 209 | 228 | 18485366 |
| YLR182W | 157 | 169 | 14998990 |
| YML007W | 5 | 59 | 11274141 |
| YMR127C | 19 | 35 | 15788653 |
| YMR239C | 461 | 466 | 15090619 |
| YNL027W | 394 | 422 | 11535618 |
| YNL027W | 612 | 615 | 11535618 |
| YOL123W | 522 | 534 | 18343812 |
| YPL153C | 785 | 807 | 15972895 |
| YPR119W | 183 | 200 | 18485366 |
| YAL040C | 559 | 580 | 11509671 |
| YBL105C | 810 | 813 | 15643058 |
| YBR098W | 244 | 263 | 14642571 |
| YCL017C | 312 | 316 | 11110795 |
| YCL067C | 1 | 13 | 1976249 |
| YCL067C | 141 | 159 | 1976249 |
| YDR034C | 190 | 250 | 10975256 |
| YEL032W | 766 | 772 | 16093348 |
| YEL061C | 994 | 1000 | 11694576 |
| YGR027C | 11 | 36 | 10386617 |
| YGR027C | 87 | 95 | 10386617 |
| YIR006C | 1024 | 1040 | 17967424 |
| YIR006C | 1145 | 1161 | 17967424 |
| YJL157C | 11 | 30 | 10485850 |
| YJL157C | 38 | 48 | 10485850 |
| YJL187C | 304 | 310 | 18562688 |
| YJL190C | 21 | 29 | 10386617 |
| YKL112W | 624 | 628 | 15522095 |
| YLR079W | 77 | 89 | 16294029 |
| YML024W | 2 | 7 | 3939318 |
| YMR036C | 31 | 33 | 18562688 |
| YOR274W | 408 | 424 | 9872948 |
