# Supplementary material for: SeqNLS: Nuclear Localization Signal Prediction Based on Frequent Pattern Mining and Linear Motif Scoring
Source: PLoS One. 2013 Oct 29;8(10):e76864. doi: 10.1371/journal.pone.0076864 (PMC3812174; doi:10.1371/journal.pone.0076864)
Supplement: Table S2 — The Hybrid NLS dataset. (DOCX) [file pone.0076864.s002.docx]

Table S2. The Hybrid NLS dataset

| UniProtKB ID | Start | Stop | Referenced PMID |
| --- | --- | --- | --- |
| IE63_SUHVK | 61 | 65 | 21795331 |
| LPIN1_HUMAN | 153 | 161 | 20660155 |
| GSTP1_HUMAN | 195 | 208 | 21782793 |
| NSA2_HUMAN | 1 | 52 | 19932687 |
| NSA2_HUMAN | 131 | 154 | 19932687 |
| Q9H2J5_HUMAN | 13 | 86 | 20056110 |
| CXCR4_HUMAN | 90 | 170 | 19796655 |
| FBX32_HUMAN | 62 | 67 | 22249105 |
| FBX32_HUMAN | 280 | 295 | 22249105 |
| CC106_HUMAN | 151 | 164 | 20159018 |
| DCAF8_HUMAN | 114 | 122 | 22500989 |
| GNL1_HUMAN | 1 | 25 | 22244851 |
| SHIP1_HUMAN | 327 | 330 | 21864674 |
| SHIP1_HUMAN | 547 | 550 | 21864674 |
| NP03_HHV11 | 190 | 194 | 21864580 |
| Q5EK48_TOXGO | 311 | 320 | 21055425 |
| E0WN99_9VIRU | 47 | 50 | 20708048 |
| DOF17_ARATH | 61 | 88 | 20116130 |
| CFLAR_HUMAN | 435 | 472 | 20016063 |
| Q95RU8_DROME | 334 | 345 | 21512259 |
| Q95RU8_DROME | 366 | 378 | 21512259 |
| Q95RU8_DROME | 407 | 419 | 21512259 |
| Q95RU8_DROME | 461 | 472 | 21512259 |
| HNRH1_HUMAN | 205 | 213 | 20308327 |
| CBY1_HUMAN | 76 | 80 | 19940019 |
| CBY1_HUMAN | 123 | 126 | 19940019 |
| SAE1_HUMAN | 192 | 210 | 21209321 |
| SAE2_HUMAN | 610 | 626 | 21209321 |
| Q869D4_SCHMA | 125 | 131 | 21220486 |
| SWI4_YEAST | 371 | 376 | 20587033 |
| NOL7_HUMAN | 1 | 10 | 20875127 |
| NOL7_HUMAN | 95 | 112 | 20875127 |
| NOL7_HUMAN | 242 | 257 | 20875127 |
| Q47867_PANAY | 97 | 112 | 21372093 |
| Q47867_PANAY | 586 | 593 | 21372093 |
| SQSTM_HUMAN | 186 | 189 | 20018885 |
| SQSTM_HUMAN | 264 | 267 | 20018885 |
| Q5I6E9_SOLLC | 504 | 510 | 20371603 |
| F6TKT8_MACMU | 657 | 781 | 20225202 |
| DAG1_HUMAN | 776 | 782 | 20512930 |
| MLXPL_RAT | 158 | 190 | 21665952 |
| PLS4_HUMAN | 273 | 280 | 21690087 |
| D2D566_MONAL | 448 | 464 | 20841357 |
| RNZ1_SCHPO | 208 | 211 | 21208191 |
| CAPSD_CFMVN | 1 | 22 | 20155311 |
| CAPSD_CFMVN | 22 | 33 | 20155311 |
| MSH5_HUMAN | 406 | 409 | 20185565 |
| NTG1_YEAST | 14 | 17 | 20194111 |
| NTG1_YEAST | 31 | 37 | 20194111 |
| PPARA_MOUSE | 71 | 100 | 22646292 |
| PPARA_MOUSE | 127 | 187 | 22646292 |
| O92424_NPVBM | 117 | 148 | 20706853 |
| NR5A2_MOUSE | 117 | 168 | 20853131 |
| NR5A2_MOUSE | 169 | 204 | 20853131 |
| SSD1_YEAST | 417 | 427 | 21762218 |
| NFE2_HUMAN | 166 | 172 | 20854373 |
| NFE2_HUMAN | 271 | 285 | 20854373 |
| TASP1_HUMAN | 197 | 220 | 21418451 |
| VPS75_YEAST | 257 | 264 | 21463458 |
| Q961C4_DROME | 347 | 353 | 20412059 |
| MSH6_HUMAN | 246 | 249 | 21437237 |
| MSH6_HUMAN | 298 | 302 | 21437237 |
| MSH6_HUMAN | 311 | 313 | 21437237 |
| SCAL_DROME | 145 | 161 | 21731746 |
| PAPS1_HUMAN | 9 | 12 | 22242175 |
| MCRS2_HUMAN | 66 | 69 | 21533551 |
| RRP4_YEAST | 122 | 151 | 20028483 |
| MEP50_HUMAN | 49 | 119 | 21789256 |
| MEP50_HUMAN | 144 | 197 | 21789256 |
| MEP50_HUMAN | 220 | 274 | 21789256 |
| I2BP2_HUMAN | 354 | 361 | 21887377 |
| O61220_CAEEL | 841 | 856 | 21957475 |
| VP16_VZVO | 302 | 347 | 21755366 |
